# Neurobiology of cognitive abilities in early childhood autism

**DOI:** 10.1002/jcv2.12214

**Published:** 2024-01-20

**Authors:** Kristina Denisova

**Affiliations:** ^1^ Division of Math and Natural Sciences Department of Psychology Autism Origins Lab City University of New York Queens College and Graduate Center Queens New York USA

**Keywords:** autism spectrum disorder, early childhood autism, early low IQ, equitable health outcomes, high IQ, infants, intelligence quotient (IQ), speech delay onset

## Abstract

This perspective considers complexities in the relationship between impaired cognitive abilities and autism from a maturational, developmental perspective, and aims to serve as a helpful guide for the complex and growing investigation of cognitive abilities and Autism Spectrum Disorder (ASD). Low Intelligence Quotient (IQ) and ASD are frequently co‐occurring. About 37% of 8‐year old children and 48% of 4‐year old children diagnosed with ASD also have Intellectual Disability, with IQ below 70. And, low IQ in early infancy, including below 1 year of age, carries a 40% greater chance of receiving ASD diagnosis in early childhood. We consider the evidence that may explain this co‐occurrence, including the possibility that high IQ may “rescue” the social communication issues, as well as the possible role of critical periods during growth and development. We consider how early low IQ may subsume a part of a subgroup of individuals with ASD, in particular, those diagnosed with autism in very early childhood, and we provide neurobiological evidence in support of this subtype. Moreover, we distinguish the concept of early low IQ from the delay in speech onset in preschool and school‐aged children, based on (*i*) age and (*ii*) impairments in both verbal and non‐verbal domains. The etiology of these early‐diagnosed, early low IQ ASD cases is different from later‐diagnosed, average or higher‐IQ cases, and from children with speech delay onset. Given recent interest in formulating new subtypes of autism, rather than continuing to conceive of ASD as a spectrum, as well as new subtypes that vary in the degree of severity along the spectrum, we identify gaps in knowledge and directions for future work in this complex and growing area.

## Graphical abstract

There are important complexities in the relationship between impaired cognitive abilities and Autism Spectrum Disorder (ASD). Available evidence suggests distinct neurobiological underpinnings and potentially distinct etiology for some of earlier‐diagnosed, lower‐IQ childhood autism cases, compared to those later diagnosed. Further, cases with early low IQ are distinguished from speech onset delay on the basis of (i) age and (ii) impairments in verbal and non‐verbal cognitive abilities. Given recent interest in formulating new subtypes of autism as well as new subtypes that vary in the degree of severity along the spectrum, this work identifies gaps in knowledge and directions for future work in this complex and growing area.

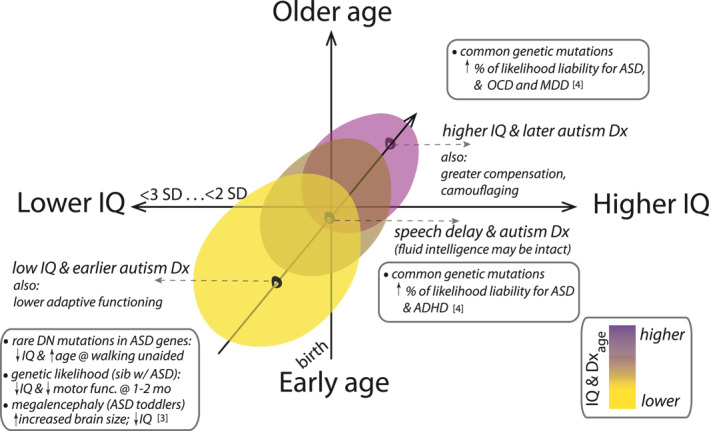

**Key points**

- There are important complexities in the relationship between impaired cognitive abilities and Autism Spectrum Disorder (ASD).
- Available evidence suggests distinct neurobiological underpinnings and distinct etiology for some of earlier‐diagnosed, lower‐IQ childhood autism cases, compared to those later diagnosed. Further, cases with early low Intelligence Quotient (IQ) are distinguished from speech onset delay on the basis of (i) age and (ii) impairments in verbal and non‐verbal cognitive abilities.
- Given recent interest in formulating new subtypes of autism as well as new subtypes that vary in the degree of severity along the spectrum, this work identifies gaps in knowledge and directions for future work in this complex and growing area.

## INTRODUCTION

**FIGURE 1:**
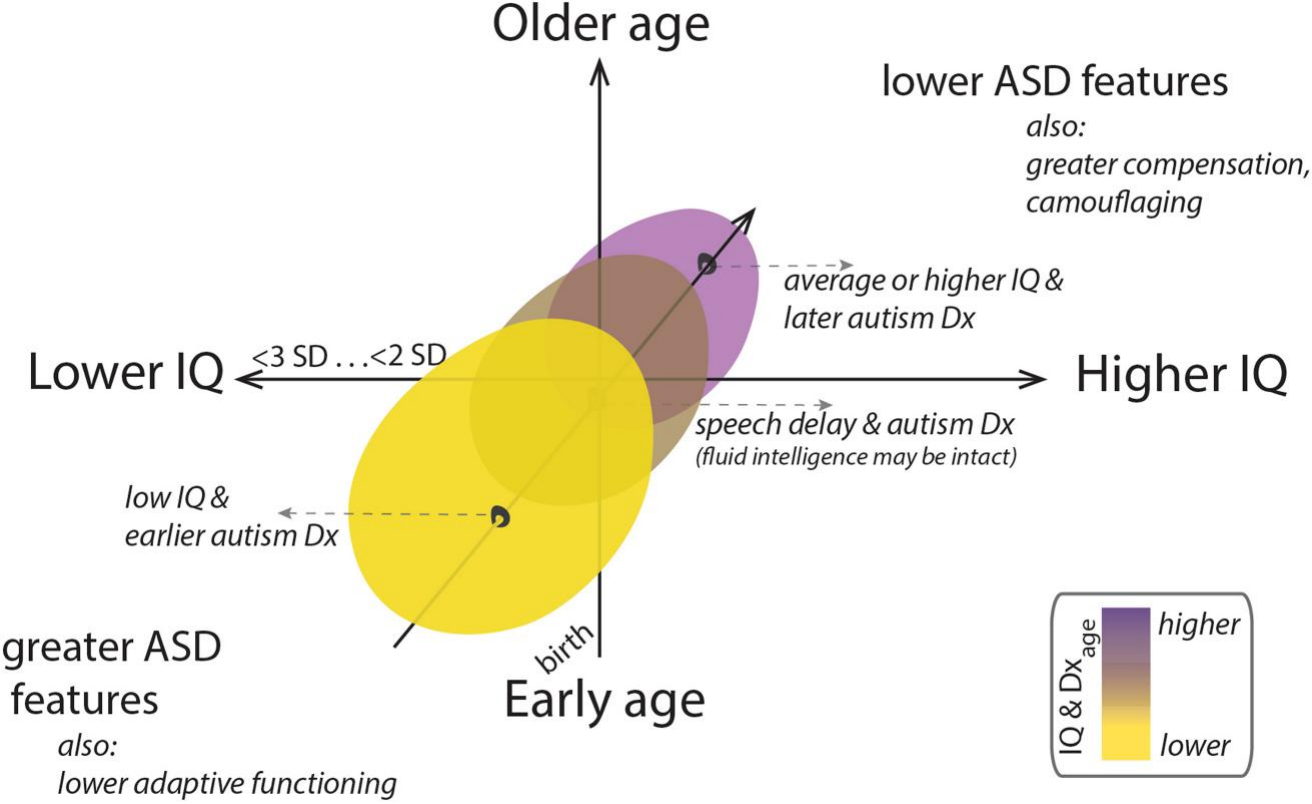
A framework presenting the relationships between autism spectrum disorder (ASD), cognitive abilities, and age. In this illustration, autism diagnosis is considered as a function of Intelligence Quotient (IQ) (lower vs. higher) and age (early infancy or toddlerhood vs. later childhood and adolescence). Lower IQ at younger ages (below about 4–5 years or even earlier in infancy and toddlerhood, below 1–2 years), as well as younger age at ASD diagnosis (i.e. initial evaluation and ascertainment during early childhood) may subsume individuals from a distinct subgroup of ASD (lower left hand region, yellow ellipse). In contrast, later‐diagnosed individuals with average or higher IQ (upper right‐hand region, purple ellipse) may subsume a different ASD subgroup. (The middle ellipse denotes individuals whose features place them midway between the two subgroupings. This group includes children with speech delay, including those cases with intact fluid intelligence. Note that low‐IQ cases are distinguished from speech delay on the basis of age and the presence of verbal as well as non‐verbal impairments.) Low IQ and ASD most likely shared early neurodevelopmental underpinnings (see text).

This perspective considers complexities in the relationship between IQ and autism diagnosis within a novel maturational framework (Figure 1). First, we clarify the neurobiological and genetic evidence that may explain the co‐occurrence between ASD and Intellectual Disability (ID) (Maenner et al., 2023; Shaw et al., 2023) and lower IQs (Denisova & Lin, 2022), including the possibility that high IQ may “rescue” the social communication impairments. Second, we consider the important role of critical periods during growth and development, how impairment of transient processes during these critical developmental windows may interact with ASD risk genes, and how these, taken together, may contribute to some early‐diagnosed ASD cases *that also have lower IQ very early in infancy*. We distinguish low IQ from speech delay onset on the basis of both age and impairments in non‐verbal as well as verbal IQ. Lastly, we identify gaps in knowledge and directions for future work in this complex and growing area. In particular, we explain how elucidating the nature of these relationships from the neurobiological perspective can affect future strategies to improve equity in early diagnosis and management of autism in underserved populations.

These goals address recent interest and important debates in understanding and formulating new autism subtypes, including an emphasis on prototypical, or representative cases (Mottron, 2021), rather than continuing to conceive of ASD as a spectrum according to DSM‐5 (Association, 2013). Broadening of the diagnostic criteria has lead to heterogeneous samples, making it difficult for researchers to discover neurocognitive mechanisms in subgroupings with shared cognitive impairments (U. Frith, 2021). Further, the Lancet Commission proposes a new subtype, “profound autism” which may subsume individuals with ASD and IQ < 50 (Lord et al., 2022). Individuals with profound autism would represent those ASD cases with the most severe manifestations by late childhood; this subtype would fit within the current spectrum framework.

Moreover, given the later‐emerging ASD cases (Riglin et al., 2021), there is further interest to investigate age at first autism diagnosis. Importantly, previous distinctions between disorders related to autism in DSM‐IV (i.e., autistic disorder, Asperger's disorder, and pervasive developmental disorder not otherwise specified, PDD‐NOS (Association, 2000)) were not based on potential differences in neurobiology (Geschwind, 2011). With regard to formulating potential new subtypes, including those subgroupings distinguished by the degree of severity as a function of age, there may be valuable differences at the neurobiological level at least for some of the cases. These differences may help inform expert clinical judgment as well as affect requirements for specific treatment and supports for autistic individuals and their families.

Herein, we introduce the concept of *early low IQ*, and clarify what is known about potential neurobiological differences in etiology of early‐diagnosed ASD cases, relative to those diagnosed in later childhood, adolescence or adulthood, and outline crucial distinctions relative to speech onset delay. This work uses both identity‐first language and person‐first language (Lord et al., 2022) and terms such as “likelihood” and “chance” whenever possible when referring to groups at “risk” for autism (i.e. to reduce potential bias, see (Bottema‐Beutel et al., 2021)). We elucidate emergent knowledge from clinical and research areas, including neurobiological findings relevant to the question of subtypes of autism, helping inform complex and growing investigations of cognitive abilities and ASD.

## OVERLAP BETWEEN LOW IQ AND ASD

Low IQ and ASD occur statistically together more often than expected by chance. This co‐occurrence is well‐established, most recently supported by the population‐based surveillance reports from the US's Centers for Disease Control and Prevention (CDC) as well as reports from other countries. About 37.9% of 8‐year old children with ASD in the US have ID, characterized by IQ less than 70 (2 standard deviations, S.D., below the population mean (Maenner et al., 2023)). These estimates are similar to those from other countries, including South Korea (33% of 7–12 year old children with ASD have IQ < 70; Kim et al., 2011) and UK (up to 55% in 10–14 year old children with ASD have IQ < 70; Charman et al., 2011). Co‐occurring ID in 7–9 year old children with ASD is detected in 12% of cases in Denmark, 39% in South‐West France, and 20% in Finland, 24% in both Iceland and South‐East France (Delobel‐Ayoub et al., 2020). A recent prevalence study from the UK with over 7 million 10‐year old school children finds that among those with ASD, about 18% have learning difficulties (including profound and multiple learning difficulties, specific learning difficulties, certain speech, language and communication needs, and sensory impairments such as hearing or visual difficulties) (Roman‐Urrestarazu et al., 2021). Estimates from even younger children indicate that about 48.5% in 4‐year old children in the US with ASD have ID (Shaw et al., 2023). These estimates represent contemporaneous levels of cognitive abilities in children diagnosed with ASD, with relatively low cognitive ability levels detected in some children—ranging from 12% to 55% —across different countries.

A recent prospective study with over 8000 infants in the US detected significantly lower IQ in children who went on to receive ASD diagnoses by early childhood (Denisova & Lin, 2022). Low IQ (<2 S.D.) during infancy, including below 1 year of age, increases the chance of future ASD diagnosis in early childhood by 40% (Denisova & Lin, 2022). Further, lower IQ in childhood significantly correlates with worse autism manifestations on the ADOS (Denisova & Lin, 2022). These findings are congruent with early smaller studies revealing lower cognitive abilities in children who are diagnosed with ASD relatively early, under 2 years of age (e.g. (Chawarska et al., 2007)). Further, more than 75% of children with early autism onset (before 3 years of age) have IQ less than 70, relative to only about 16% of children with late onset ((Kolvin et al., 1971); also see (Gittelman & Birch, 1967); cf. (Carr, 1976)). Taken together, these data pose a question as to *why some children with lower IQ might be diagnosed early (*vs. *late)*? Another question might be: *What are some important implications of these data*? The following sections consider several, not necessarily mutually exclusive, possibilities.

## NEUROBIOLOGY UNDERLYING OVERLAP BETWEEN IQ AND ASD

At the neurobiological level, both lower IQ and an ASD diagnosis may have a common genetic basis (Parikshak et al., 2013). A combination of both low IQ and autism diagnosis is consistent with functionally deleterious impacts of rare de novo (DN) variants of large effect associated with both ID and ASD (Myers et al., 2020). As well, deleterious impacts of multiple DN likely gene disrupting (LGD) mutations of small effect could also produce both low IQ and ASD manifestations (Ronemus et al., 2014). These features most likely shared early neurodevelopmental underpinnings.

In particular, Satterstrom and colleagues conducted the largest whole‐exome sequencing study to date characterizing rare DN and inherited coding variation in 11,986 individuals with ASD, and identified 102 high‐risk ASD genes affected by the variants. These genes were further categorized into ASD‐predominant (P) genes and ASD‐neurodevelopmental disorder (NDD) genes (Satterstrom et al., 2020). The authors detected a significantly lower IQ (a reduction by almost 1 S.D.: 11.6 points) and lower age of walking unaided (representing a motor delay of 2.6 months) in ASD individuals with disruptive DN variants in ASD‐NDD genes relative to ASD‐P genes. This genetic evidence points to an extraordinarily early developmental interaction between processes contributing to ASD as well as impaired IQ and early sensorimotor functioning. Further, lower IQ and delay in walking onset was observed in ASD cases with variants in both sets of ASD risk genes, those predominantly ASD and ASD‐NDD genes, relative to ASD cases without DN variants in these genes (Satterstrom et al., 2020). Overall, this evidence establishes that as a group, ASD with rare DN variants can be distinguished by functional severity—by lower IQ level and impaired sensorimotor functioning—from ASD without DN variants.

**FIGURE 2:**
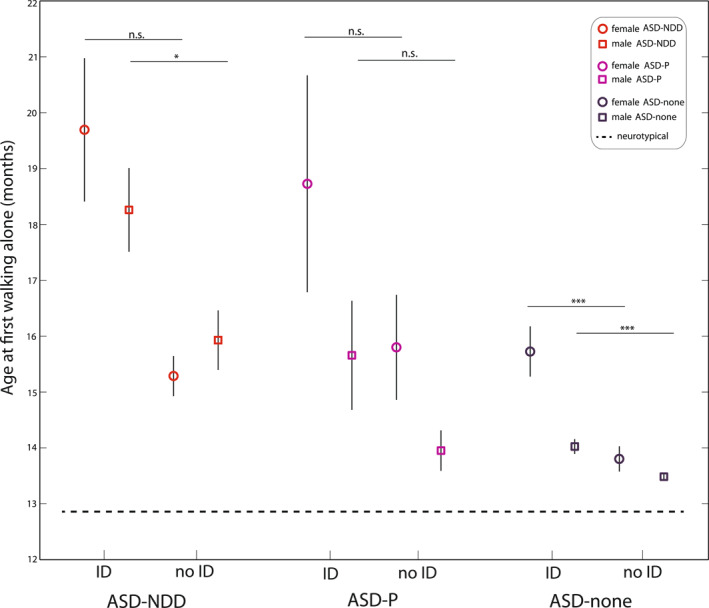
Subgroupings of autism spectrum disorder (ASD) cases with variants on ASD‐NDD and ASD‐P genes and those without (i.e., ‘ASD‐none’), by intellectual disability category and by males and females, vis‐à‐vis age at first walking alone (S4 Table in Satterstrom et al. (2020)). Neurotypical baseline (age at first walking alone, 12.9 months) is from Ertem et al., 2018. All *t*‐tests two‐tailed. (*: *p* < 0.05; ***: *p* < 0.001; n.s.: not significant.) Plotted are means with s.e.m bars (+/− s.e.m., standard error of the mean).

Given the higher ratio of males versus females with ASD in the population (male‐to‐female prevalence ratio is 3.8 (Maenner et al., 2023)), the association between sensorimotor functioning and IQ is further explored using S4 Table data from Satterstrom et al. (2020). Forming subgroupings by ID category (under ‘phenotype’ in S4 Table), we can see that females with ID and a variant on the ASD‐NDD genes have the oldest age at first walking relative to all other subgroupings (Figure 2). These females start to walk on average at 19.7 months relative females without variants and no ID (∼13.8 months)—a delay of 6 months (*t* (383) = 6.48, *p* = 2.7695e‐10). Males with ID and a variant on the ASD‐NDD genes start to walk at 18.26 months relative to males without variants and no ID (a delay of almost 5 months; *t* (2436) = 9.4797, *p* = 5.8288e‐21). Within the ASD‐NDD group, females with ID have a 4.5 months delay relative to those without ID (*t* (31) = 1.76, *p* = 0.08); the delay in males is about 2 months, *t* (90) = 2.45, *p* = 0.02. Within the ASD‐P group, females with ID have a 3 months delay relative to those without ID (*t* (19) = 1.31, *p* = 0.2); the delay in males is about 1.7 months, *t* (96) = 1.95, *p* = 0.05.

It is interesting to observe that in ASD cases without variants on either ASD‐NDD or ASD‐P genes (‘ASD‐none’ subgroup’), females with ID still have a delay of about 2 months relative to those without ID (*t* (592) = 4.19, *p* = 3.2398e‐05) whereas the difference in males with and without ID is only about 2 weeks (*t* (3459) = 3.89, *p* = 1.0345e‐04) (all tests two‐tailed). This large and significantly greater delay in age at walking in female probands who do not harbor variants on these genes suggests that the biological basis of autism in females with ID and early sensorimotor impairments may be underwritten by additional biological factors other than DN mutations, by non‐biological contributions from the environment, or via gene × environment interactions (Schaafsma & Pfaff, 2014) to which female infants are particularly vulnerable.

Here, about 9% of ASD cases have disruptive variants on these specific genes (Table S4 (Satterstrom et al., 2020)). Overall, it is estimated that DN mutations (copy number variants (CNVs), LGD variants, and missense variants—combined on the assumption of additivity (Iossifov et al., 2014)) occur in about 28% of autism simplex cases (defined as one child with autism in the family) (Iossifov et al., 2014).

In addition to examining the contribution of specific genes that are targets of mutations, another approach is to examine the effects of different types of variants on the genome. Recently, Douard et al. (2021) examined the functional effects of the two types of CNVs, deletions and insertions, and found that the effect of deletions is more deleterious on cognition and behavior. Decrease in IQ by 2.6 points was associated with each deleted point on the pLI metric (probability of being loss‐of‐function intolerant), with smaller effect on IQ from duplications (Douard et al., 2021). While both deletions and duplications were associated with a delay in the first age at walking alone (by 5.46 and 3.58 months, respectively), the association was driven by lower non‐verbal IQ in those with deletions (Douard et al., 2021). This study further supports the strong link between IQ, early sensorimotor development, and genetic liability.

We next consider evidence from studies linking genetic likelihood for ASD with sensorimotor, cognitive, and socio‐cooperative impairments in infants who do not yet have ASD diagnoses, but who are at heightened familial likelihood (Bottema‐Beutel et al., 2021) for ASD.

Newborns with an older biological sibling with an ASD are themselves at a heightened genetic likelihood (HL) for developing an ASD (recurrence likelihood ∼4% to 17% (Palmer et al., 2017)). A number of HL infant studies report evidence on possible disruptions of early basic social and attentional mechanisms and lower intelligence (e.g. (Denisova, 2019; Denisova & Zhao, 2017; Jones & Klin, 2013)). In a set of quantitative studies examining sensorimotor and cognitive functioning, Denisova and Zhao (2017) show that as early as 1–2 months of age, differences in sensorimotor functioning already distinguish HL from low likelihood (LL) infants. As an early precursor of socio‐cooperative impairments characteristic of ASD, HL infants' head movements are similar (rather than different, as in LL infants) when resting or sleeping, compared to listening to a biologically important stimulus, the human voice during an fMRI scan (Denisova & Zhao, 2017). Moreover, HL infants in that sample have lower IQ (on Mullen Scales of Early Learning, MSEL (Mullen, 1995)) relative to LL infants (Denisova & Zhao, 2017). The link between lower IQ (including both verbal and non‐verbal components) and HL status is further supported with data from 1445 infants from HL infant sibling studies across the US and UK. Specifically, LL infants have a consistently higher IQ relative to HL infants, including those who converted to an ASD (Suppl. Figure 4 in (Denisova & Zhao, 2017)). In infant populations at a heightened genetic, biological likelihood for ASD, these results further reveal an association between early atypical social information processing and a lower IQ.

It can be noted that neurogenetic conditions known to associate with both ASD and ID include Fragile‐X and Tuberous Sclerosis Complex, and such Mendelian forms of ASD have penetrance of less than 50% for ASD (Geschwind, 2011). Moreover, these “syndromic” types account for only about 1% of ASD cases (Geschwind, 2011). This low estimate is in contrast to the recurrence likelihood for ASD in up to 17% of infants at HL for ASD from a population‐based study (Palmer et al., 2017), and may be higher (e.g. up to 25% (Ozonoff et al., 2011)).

## EARLY LOW IQ

Taking together findings on the genetic association between the older age at walking and lower IQ in ASD cases with rare DN variants in ASD‐NDD and ASD‐P risk genes (Satterstrom et al., 2020), sensorimotor and cognitive evidence from 1 to 2 months‐old infants who are merely at a high genetic likelihood for ASD (Denisova & Zhao, 2017), and significantly increased chance for ASD in children from the general population who had lower IQ in infancy (Denisova & Lin, 2022), we may consider how lower IQ may subsume a part of a ***subgroup of individuals with ASD***, in particular, those diagnosed with ASD in very early childhood.

This scenario would suggest a **putative subtype** of early childhood‐diagnosed autism, that is, **lower IQ essentially coupled to ASD** (Denisova & Lin, 2022). Here, the time frame with regard to age since birth is relative; “early” means from birth to *before* 4–5 years of age, and “very early” means even earlier, from birth to *before* 2–3 years of age. The concept of early low IQ (LIQ) is defined as overall cognitive ability assessed relatively early in life, with scores falling at least 2 S.D. below (<70) the population mean. Early extremely low IQ is about 3 S.D. below the mean (<55). Early lower IQ is only about 1 S.D. below the mean (<85).

Early low IQ is distinguished from delay in speech onset by (*i*) age, and by (*ii*) impairments in non‐verbal as well as verbal domains. The concept of early (*below* 4–5 years) or very early (*below* 1 or 2 years) low IQ is concerned with a critical time period very soon after birth (discussed below), when both non‐verbal and verbal impairments increase the chance for future ASD outcomes. Several illustrative examples from non‐verbal and verbal domains are provided, as follows.

For instance, a non‐verbal, Fine Motor probe on the MSEL, related to grasping objects, (‘reaching and grasping block (not reflex)’) is a milestone that is generally reached around 3.5 months of age (see item #82 in (Ertem et al., 2018)). Another non‐verbal Visual Reception probe on the MSEL, checking whether the infant actively looks for toys or objects that disappear (‘looks for toy covered, then displaced’) is a milestone reached around 6.3 months (item #89 in (Ertem et al., 2018)). Moreover, a verbal Receptive Language probe on the MSEL related to responsiveness to social sounds (‘responds to voice and face by vocalizing’), is reached around 1.6 months (item #29 in (Ertem et al., 2018)). As well, a verbal Expressive Language probe on the MSEL, related to communication (regarding whether the infant ‘plays gesture/language game’) is reached around 5.4 months (item #6 in (Ertem et al., 2018)).

These milestones are all reached *before* the 1st birthday in neurotypically developing children worldwide (Ertem et al., 2018). Infants below 1 year of age who scored 2 SD below standardized full‐scale IQ had a 40% greater chance of receiving ASD diagnoses by the time they reached early childhood (Denisova & Lin, 2022) and findings were consistent when data were examined separately by non‐verbal and non‐verbal domains. Note that these age‐appropriate developmental probes rely on relatively intact motor functioning and prompt a behavioral motor response to social sounds (for example, spontaneous vocalizing, which is a motor act, or head turning). That is, the idea that motor functioning in infancy, especially before 2 years of age, is unimportant for cognition is rejected. Indeed, nascent sensorimotor functioning is linked to future cognitive development in infants and a lack thereof to atypical cognitive development (e.g. (Denisova, 2019; Denisova & Zhao, 2017)).

**FIGURE 3:**
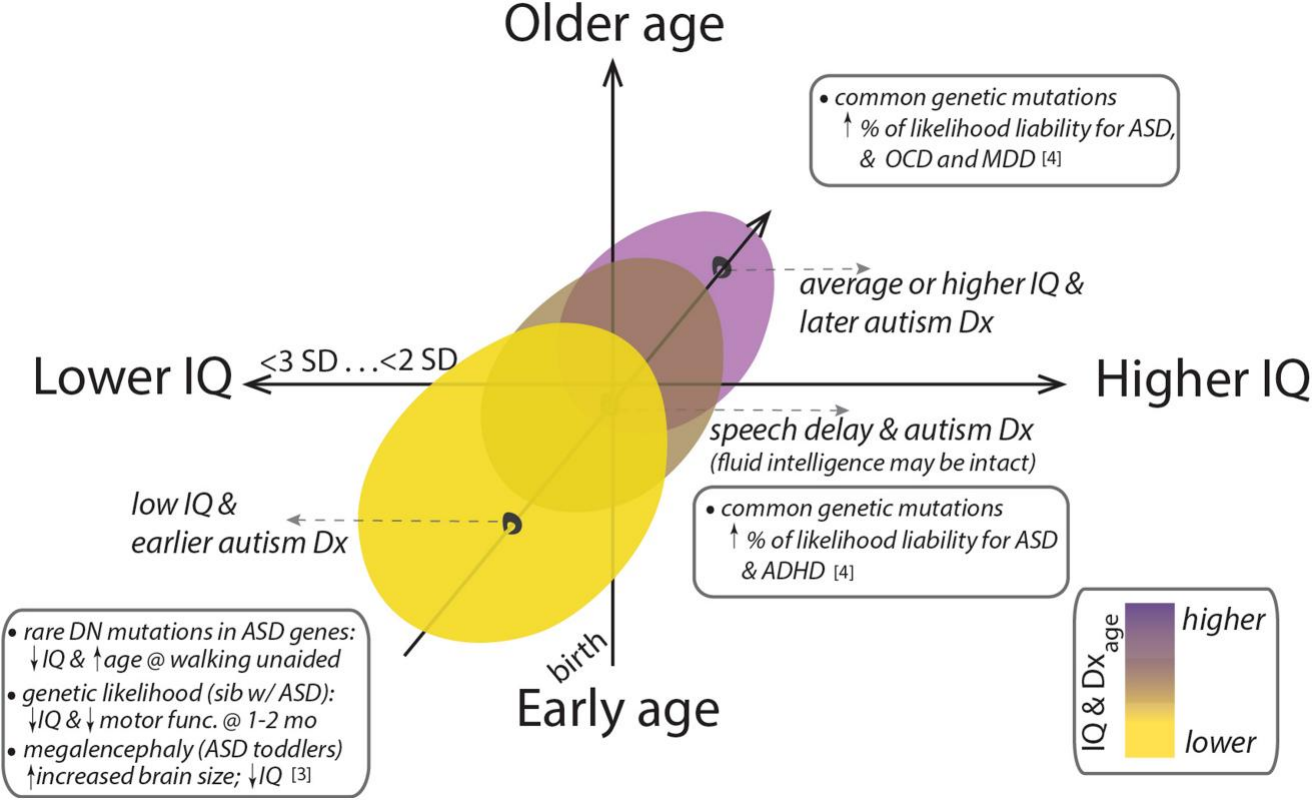
Elaboration of the autism subgroupings based on age and cognitive abilities with neurobiological evidence. The subgroupings in this framework are motivated by several lines of converging evidence, including from epidemiological studies indicating a significant association between ID and ASD, especially in early childhood, prospective studies revealing prognostic value of early low IQ for childhood autism (early‐diagnosed children who have had low cognitive abilities *before* their diagnoses), and genetic studies detecting overlap between rare DN variants in genes in individuals with ASD and those with ID. Age is an important factor because critical periods occur uniquely during prenatal, neonatal, and early childhood time frames. For this reason, disruptions in genes contributing to both lower‐IQ and ASD may interact with transient developmental processes, including important sensorimotor, socio‐cooperative, and cognitive processes, and contribute to autism that is characterized by early low IQ. Taken together, the evidence suggests distinct neurobiological underpinnings and potentially distinct etiology for some of earlier‐diagnosed, lower‐IQ childhood autism cases (lower‐left quadrant), compared to those later diagnosed (upper‐right quadrant). For example, ASD manifestations of later diagnosed, higher‐IQ individuals may be underwritten by common mutations or variants (e.g. rather than rare DN mutations as for lower‐IQ individuals) and these individuals may also have different comorbidities relative to early‐diagnosed autism with lower IQ. Individuals within the lower‐right quadrant, who have speech delay but who may have intact fluid intelligence, may also have additional learning‐related comorbidities such as ADHD; ADHD is predominantly underwritten by common, rather than rare mutations. ADHD, Attention Deficit/Hyperactivity Disorder; ASD, Autism Spectrum Disorder; DN, de novo; ID, Intellectual Disability; IQ: Intelligence Quotient; MDD: Major Depressive Disorder; OCD: Obsessive Compulsive Disorder. References [1] Satterstorm et al., 2020; [2] Denisova & Zhao, 2017; [3] Lee 2021; [4] Gandal 2016.

The delay of speech onset, such as lack of phrase speech, is identified somewhat later in childhood. The milestone of using phrases with least 3 words to communicate is reached around 2 years of age worldwide (24.9 months, item #19 (Ertem et al., 2018)). In research with minimally verbal children, a benchmark of 5 years has been noted as significant for prognosis, as after the age of 5 the acquisition of substantial language skills is unlikely (Tager‐Flusberg & Kasari, 2013). Some children with ASD and language delay (especially those with impaired expressive speech) may have intact non‐verbal IQ or normal fluid intelligence, and represent a unique, separate subgroup (middle ellipse in Figures 1 and 3) relative to the early (or very early) low IQ group. They may have distinct neurobiology relative to early low IQ and later‐diagnosed, higher IQ cases, although there is overlap between all groups. To distinguish children with speech delay from these other groups is particularly challenging, and important concerns are discussed in greater detail in the last section on future research directions.

Overall, reduced IQs during infancy and very early childhood may not “rescue” children from the most severe and functionally maladaptive consequences of the genetic aberrations. Infants or toddlers with diminished IQ or cognitive abilities and more severe autism manifestations might also have a clear impairment (i.e. with frank manifestations), and thus may be likely recognized and diagnosed at a younger age. That is because a clear impairment such as lack of communication and eye contact during early infancy and toddlerhood would be expected to raise concerns with parents, caregivers, and medical professionals.

Further, the role of adaptive functioning in children with ASD relative to cognitive ability levels is significant and continues to be rigorously investigated. In studies with children from the general population, Charman and colleagues found lower adaptive functioning relative to IQ in children with ASD (Charman et al., 2011). In infants at HL for autism, Bussu et al. (2019) and Zwaigenbaum et al. (2021) detected an association between lower IQ and lower adaptive functioning (Bussu et al., 2019; Zwaigenbaum et al., 2021). Consistent with these findings, children with ASD who received the highest IQ scores also had significantly higher adaptive functioning, relative to typically developing (TD) children with the lowest IQ scores who had lower adaptive functioning (Denisova & Lin, 2022). An important possibility is that higher IQ scores may facilitate a child's attempts to “blend in” within their surroundings (Livingston, Colvert, Bolton, & Happé, 2019).

## THE POSSIBILITY THAT HIGHER IQ MAY “RESCUE” SOCIAL COMMUNICATION ISSUES

A child with higher cognitive abilities and few or less severe symptoms may not raise similar concerns with parents or caregivers, or concerns might be raised much later, in adolescence or adulthood, relative to a child with lower cognitive abilities. The higher‐IQ may “rescue” the developing brain during early childhood, with ASD diagnosis ascertained later in adolescence or adulthood, in some children. There may be important differences in comorbidities in higher‐IQ, later diagnosed ASD cases (vs. early‐diagnosed children). Higher‐IQ children with ASD may have internalizing symptoms (e.g. anxiety (Livingston, Colvert, et al., 2019)) because they may be compensating or camouflaging their condition. Compensation involves “alternative cognition to circumvent underlying cognitive difficulties” (Livingston, Colvert, et al., 2019). The process of compensation is more cognitively demanding than camouflaging, which may involve more superficial behavioral modifications in an attempt to “blend in” (Livingston, Shah, & Happé, 2019). For example, compensation may involve specific rules for behaving in familiar but still uncertain situations. However, while high camouflagers or compensators have better social engagement and communication (Corbett et al., 2021), compensation also has a cost for the autistic individual ((Uta Frith, 1991), p. 21). Livingston et al. (2019) found that in autistic adolescents with theory of mind impairments, high compensation is associated with higher IQ, Executive Function, and social skills, but also significantly greater anxiety obtained via self‐report. As parental ratings of anxiety did not differ significantly among high and low compensators, anxiety may be underestimated in those with high compensation (Livingston, Colvert, et al., 2019). Crucially, parents of higher‐IQ children may underestimate anxiety in their child (Livingston, Colvert, et al., 2019). Thus, diagnosis at a later age for some average or higher‐IQ children may be due to compensation strategies (which may make it harder for parents to assume that there is an impairment in communication) as well as potential unawareness of parents that anxiety or depression is a sign of concern and may be related to autism.

In later‐diagnosed, average or higher‐IQ ASD cases, the genetic likelihood may be due to common genetic mutations or variations which are known to occur in a portion of individuals with ASD, as well as those with Obsessive Compulsive Disorder and those with Major Depressive Disorder (Gandal et al., 2016). It may also be due to the variants accruing via transmission from the parents (rather than arising DN) or due to non‐genetic contribution (Iossifov et al., 2015). Some children with ASD and speech delay onset may harbor common genetic mutations related to atypical learning, similar to those with attention deficit/hyperactivity disorder (ADHD) (Gandal et al., 2016). As noted above, this is in contrast to lower‐IQ ASD cases, whose neurobiological basis may be underwritten by rare DN mutations and which account for a large proportion of likelihood liability for both ASD and ID (Gandal et al., 2016). **Herein we primarily focus on lower‐IQ, early‐diagnosed ASD children and potential neurobiological underpinnings of early ASD diagnosis related to atypical developmental processes.** The etiology of these early‐diagnosed, lower IQ ASD cases is likely to be different from later‐diagnosed, average or higher‐IQ cases, and school‐aged children with speech delay onset, which are further acknowledged in the last section outlining directions for future work. As considered below, many of the early‐diagnosed ASD cases likely have an atypical biological process underpinning the development of the nervous system, specifically related to the interactions of emergent ASD symptoms, lower IQ, and atypical unfolding of developmental processes during critical periods.

## THE ROLE OF CRITICAL PERIODS IN EARLY LOW IQ AUTISM

The critical periods during prenatal as well as postnatal growth and maturation are highly important, but presently somewhat under‐recognized, for their significant role in the etiology of early‐diagnosed, lower‐IQ ASD. Critical periods subsume many unique processes driven by genes that are temporally and spatially regulated (Kang et al., 2011). For example, there are temporal differences in expression trajectories of genes coding for synapse and dendrite development, as well as differences in trajectories across different brain regions (e.g. the neocortex vs. amygdala vs. the cerebellum) (Kang et al., 2011). With regard to early autism manifestations, disruptions of genes supporting normative brain development (Silbereis et al., 2016) could interact with disruptions in ASD risk genes. Conversely, mutations in ASD‐associated genes may directly disturb “intersecting developmental processes” (Willsey et al., 2013). Interestingly, a few genes (e.g. GABRB3, Gamma‐Aminobutyric Acid Type A Receptor Beta3 Subunit, discussed below) are critically involved in early developmental processes as well as have been identified as ASD risk genes (Satterstrom et al., 2020).

## ACQUISITION OF NATIVE LANGUAGE DURING THE FIRST YEAR OF LIFE

One of the developmental processes that requires both sensorimotor functioning and socio‐cooperative cognition during the 1st year of life is first language acquisition (termed “vocal learning” in songbirds (Pfenning et al., 2014)). It requires active listening, functioning motor circuits, and a social tutor (Denisova, 2019). Because production of sounds such as babbling in humans or “subsong” in juvenile songbirds (vs. simply listening) is a motor act, the presence of motor activity drives functional changes in neurons (Jin & Clayton, 1997), via gene expression by immediate‐early genes (e.g. EGR1). This plasticity process contributes to synapse changes and circuit development (Hayase et al., 2018), and gradually declines with age (Jin & Clayton, 1997).

When unfolding appropriately during maturation in human infants, the process of language acquisition involves precise reorganization of cortical, including sensorimotor, and subcortical circuitries (Denisova, 2019) and enables the “tuning in” to one's native language by about 9 months of age (Kuhl, 2004). It provides scaffolding for additional, more complex social interactions (Denisova, 2019). Impairments in genes supporting this and related early‐life mechanisms may have cascading effects for downstream, more complex circuit development and thus could underlie the etiology of some of the lower‐IQ, early‐diagnosed ASD cases. There is empirical support for this possibility, as follows.

Intriguingly, GABRB3 (and GABRB2) are two protein‐coding genes identified by Pfenning et al. (2014) as vocal learning genes with convergent genetic expression in both songbirds and humans. Both are also among the 102 ASD risk genes identified by Satterstrom et al. (2020) and classified as “neuronal communication” genes (Satterstrom et al., 2020). (Note, GABRB3 was identified by Iossifov and colleagues (Iossifov et al., 2015) to have a low LGD (likely gene disruptive) score of 0.08 (<0.1) (scores range between 0 and 1), indicating that a DN mutation on that gene is particularly likely; GABRB2 has an LGD score of 0.12.) In addition to ASD, GABRB3's disruption has been implicated in other neurodevelopmental disorders (epilepsy (Epi4K & Investigators, 2013)). Further, what is unique about the GABRB3 (and GABRB2) is that while it codes for an inhibitory neurotransmitter GABA in adults, GABA actually has a transiently *excitatory* role during early development (e.g. (Valeeva et al., 2016)).

Improved understanding of how mutations in genes including GABRB3 and other genes related to motor functioning (e.g. SHANK3) act in concert during early development would provide insight on early morphological disruptions of maturing brain circuitries during neonatal and infant periods and is especially pertinent for ASD (Lee et al., 2021). Additional research may also help inform debates on the excitatory‐inhibitory (E/I) ratio disruption in ASD. From the perspective of the E/I ratio imbalance in ASD as a compensatory mechanism (Antoine et al., 2019), it would be informative to understand how the mechanism would account for upstream perturbations occurring during the critical windows in the neonatal period, compared to later in childhood.

## MAPPING TO EARLY BRAIN CIRCUITRIES

How early‐life disturbances at the genetic level might manifest in atypical brain circuitries, lower IQ and early ASD manifestations is still a matter of intense research. Based on current knowledge, many different but also interacting genetic and molecular pathways underlying early ASD manifestations could be involved (for review, see (Geschwind, 2011)), including those with broad and variable cognitive effects. For instance, atypically developing pathways related to circadian, sleep, and sensorimotor development could have putative knock‐down effects on memory consolidation and learning during early life (Rasch & Born, 2013). Atypically developing synaptic pathways could impair brain circuitries and lead to impaired cognition (Zoghbi & Bear, 2012). Megalencephaly (enlarged brain growth) and lower IQ have been observed in toddlers diagnosed with ASD in early childhood (Lee et al., 2021). Moreover, atypically developing limbic pathways could lead to increased anxiety and self‐regulatory problems due to inability to infer intent in complex, unpredictable social interactions (Denisova et al., 2016; Denisova et al., 2013). There is also an important role for gene‐environment interactions (Chaste & Leboyer, 2012), including contributions of genetic vulnerability to in‐utero exposure to medications (Denisova, 2021). In particular, epigenetic processes play a role in the emergence of ASD (Schaafsma & Pfaff, 2014). The idea that very different genetic and molecular pathways could converge onto common structural problems in the developing body is supported by research in the field of congenital heart defects (CHDs). The CHDs may be caused by very different defects in different genes and also expressing very differently phenotypically (Myers et al., 2020; Pierpont et al., 2018). Importantly, regardless of the underlying genetic mechanism, cognitive impairments very early in life may be considered a key intermediate link for predicting some of the early‐diagnosed ASD cases. Figure 3 presents the framework together with some of the available neurobiological evidence, and with the distinctions elaborated in greater detail, and **Key Concepts** presents important considerations for ongoing and future research.

## LIMITATIONS AND IMPORTANT CONSIDERATIONS FOR FUTURE RESEARCH

By school age, minimally verbal children with ASD remain understudied (Tager‐Flusberg & Kasari, 2013). Researchers agree about the great heterogeneity characterizing this overall group (Tager‐Flusberg & Kasari, 2013): while some children are impaired on both expressive and receptive language, others with few expressive abilities have intact receptive abilities, and not all have low non‐verbal IQ. It is noted that children who acquired some language after age 5 have non‐verbal IQ over 50 (Tager‐Flusberg & Kasari, 2013). This conclusion is consistent with a study of school‐aged children (average age 11.6 years) with ASD who reportedly did not attain phrase speech by 4 years of age Wodka et al. (2013). These children's higher non‐verbal IQ is strongly correlated with later speech acquisition (Wodka et al., 2013), supporting the importance of non‐verbal IQ for later development. Additional studies are needed with developmentally delayed children who may have had low IQ or low verbal IQ in infancy but who are not diagnosed with an ASD. There may be a separate group of children who have delayed speech (i.e. who are ‘late talkers’), but who catch up to their peers.

One important concern is that standardized observational instruments assessing cognitive abilities measure characteristics of performance (i.e. what infants or toddlers actually “*do*”), not competence—which is *inferred* from their observable behavior. Some minimally verbal children with ASD may have cognitive abilities in the normal range, but the norm‐based instruments used to assess their abilities inadvertently place disproportionate demands on these children who may not be able to comply (e.g. requiring motor activities for responding, such as pointing). Specifically, some school children with language delay or minimal language may be at risk for being underestimated on their cognitive abilities (Courchesne et al., 2015). To assess cognitive abilities in these children, it may be necessary to use a strength‐based assessment which obviates verbal or explicit instructions and does not require an overt response (e.g. pointing, a motor response) (Courchesne et al., 2015). That is, some children with speech delay are nonetheless responsive to assessments that do not rely on language.

For example, in a pilot study more than 50% of children ages 6–12 with minimal spoken language achieved an IQ score of 75 or higher on the Raven's Colored Progressive Matrices board form (Courchesne et al., 2015) (This version of the Raven was chosen because it does not require language comprehension or production, or pointing (Courchesne et al., 2015).) Especially for this group of children, it would be important to take into account factors related to measuring of intelligence, including discrepancy between verbal and non‐verbal estimates (Girard et al., 2021) and additional factors, including inability to achieve a basal score (Courchesne et al., 2015). Fluid intelligence may be unaffected or even superior in some school‐aged children with ASD who are minimally verbal, despite seemingly unestimable (or low) general intelligence assessed using traditional instruments (Courchesne et al., 2015; Dawson et al., 2007). Further, some preschoolers diagnosed with ASD who have atypical language development may have a speech sound disorder, as in a case of one HL infant who received ASD diagnosis but was later ascertained to have a speech problem and their ASD diagnosis deemed a false positive (Camarata, 2014). However, even when strength‐based assessments are used, about 43% of children still score below IQ of 75 (Courchesne et al., 2015), confirming low general cognitive abilities in some minimally verbal ASD children. While minimally verbal, these children's impairments are not restricted to the verbal domain; they have impairments in the non‐verbal domain as well.

The question of distinguishing children with low IQ versus those with speech delay or low VIQ alone does not arise in very early infancy or early toddlerhood. For infants below 1 or 2 years of age—those who have very early low IQ—this time period is before the 2‐year milestone when phrase speech is normally achieved. Therefore, information about low IQ (both verbal and non‐verbal) of infants below 2 years of age can be used in a predictive manner for future ASD outcomes, with 40% greater likelihood of very early low IQ predicting future ASD outcomes (Denisova & Lin, 2022). Moreover, cross‐cultural evidence suggests that specific milestones, including milestones on relating to other people during the 1^st^ year of life, are relatively independent of language ability and are stable across different cultures (Ertem et al., 2018).

For toddlers below 4–5 years of age, research shows that IQ is still lower for those with ASD versus non‐ASD outcomes (including those with language delay). Early low IQ *before* preschool heralds future atypical outcomes, regardless of whether it might later manifest as an ASD (in 40% of cases) or as another developmental disability including general or language delay, or as a learning disability (Denisova & Lin, 2022). Specifically, the 3^rd^ group of infants who had ASD ruled out but who had another developmental outcome (i.e. language delay or general delay) also had significantly lower IQ (full‐scale, verbal, as well as performance) relative to TD children; the ASD group still had the lowest IQ of the three groups (Denisova & Lin, 2022). This finding suggests that one early factor distinguishing *future* non‐ASD delay (including language delay) from ASD per se is the severity of both non‐verbal and verbal cognitive abilities: these are less impaired for children with non‐ASD delays.

However, some of the preschool (and older school‐aged) children with speech delay with or without ASD may have low VIQ (either low expressive language, or low expressive and receptive language) while PIQ or fluid intelligence as estimated using strength‐based instruments may be at normal levels. Particularly for children between 2 and 5 years of age, additional research and careful differential ascertainment (Camarata, 2014) and testing (Courchesne et al., 2015) are needed to distinguish children with speech delay from those with low IQ and no speech delay. Additional studies are needed with developmentally delayed children who may have had low IQ or low verbal IQ in infancy but who are not diagnosed with an ASD.

Another way to distinguish between these important subgroups is to use brain imaging as a non‐invasive tool to assess developing circuitries that underlie cognition in children, before differential diagnosis and to help inform clinical judgment. New research is needed to study young children's brain structure and function using task‐free, non‐invasive brain imaging techniques, in particular Magnetic Resonance Imaging, a tool which is able to image subcortical brain structures and does not require overt motor response or understanding verbal instructions. Very little is known about the neurobiology distinguishing these children from very early low IQ infants or from those with higher‐IQ who are diagnosed in adolescence or adulthood. One possibility is that children with ASD who have speech or language delay and learning difficulties (and whose fluid intelligence is intact) may have overlapping etiology with those children who have ADHD. Some of these children's phenotype may be due to common genetic mutations (Gandal et al., 2016). This mechanism is in contrast to very early low‐IQ infants who go on to receive ASD diagnoses as noted earlier, those whose low VIQ and PIQ is due to very early (below 1 year of age) developmental impairments in sensorimotor and vocal learning circuitry, which would impact both verbal and non‐verbal domains.

Understanding the neurobiology underlying sex differences in very early life sensorimotor development in female infants who go on to develop ASD, relative to non‐ASD delay (e.g. speech delay) should be a priority. While ASD females with ID and with variants on ASD‐NDD genes had the oldest age at walking, it is worth highlighting that among ASD females without variants on ASD‐NDD genes, those with ID still had significantly higher age at walking relative to those without ID (see Figure 2). New studies are needed to understand the biological basis of low IQ in female infants from the general population who go on to receive ASD diagnoses, and to distinguish how females within this group (i.e. (very) early low IQ) differ from others (e.g. higher‐IQ) in neurobiology.

While children with lower IQ and more severe autism are more likely to be diagnosed earlier, diagnosis depends on access to autism evaluation and availability of services, and such important access is limited in some communities. In underserved populations, early diagnosis and treatment are negatively affected by complex racial‐ethnic and socio‐demographic, including immigrant, factors (i.e. (Durkin et al., 2010; Kelly et al., 2019; Khanlou et al., 2017; Yingling & Bell, 2019; Yingling et al., 2022)). There are racial‐ethnic and neighborhood inequities in the age of treatment receipt (Yingling & Bell, 2019). Relatedly, there is both unequal access to services and therapists (Yingling et al., 2022) and inequitable utilization of services, with a smaller proportion of Black and Latino as well as low socio‐economic status children receiving services (Yingling et al., 2019). In particular, among children with IQ < 70 and ASD diagnosis, there is a 6‐month lag in the age at first autism diagnosis for African American/Black infants reported in (Maenner et al., 2020). This delay is concerning as African American as well as Hispanic children *with* ASD are more likely to have ID relative to White children (50.8%, 34.9%, and 31.8%, respectively (Maenner et al., 2023)).

One goal for future work is to more effectively leverage the strong evidence that early cognitive, socio‐cooperative, and sensorimotor problems herald ASD manifestations. Specifically, it would be important to further explore ways to disseminate objective, neurobiologically‐grounded information that some of the early drivers of autism are due to biological processes to both parents and providers in the communities. Providing such objective neurobiological information might help remove some of the stigma related to autism diagnosis in the underserved communities (e.g. (DiGuiseppi et al., 2021; Khanlou et al., 2017)) and facilitate earlier diagnoses.

As well, further research is needed to study genetic contributions to ASD cases diagnosed later in life (e.g. (Riglin et al., 2021)), and which may have average or higher‐IQ. Later‐diagnosed ASD cases are already noted to have different comorbidities (e.g. anxiety, internalizing symptoms, etc.) and are likely to have different etiology relative to early‐diagnosed ASD cases. Relatedly, children and adolescents with ASD and highly specialized skills or “savant” abilities, such as absolute pitch (Mottron et al., 2013) may have atypical neurocognition. More studies are needed to understand the neurobiological underpinnings of these later‐diagnosed, higher‐IQ ASD. This goal is also particularly important for advancing equitable health outcomes, as it has been noted that both lower‐IQ and higher‐IQ children from underserved populations are at risk for under‐diagnosis (Fombonne & Zuckerman, 2022).

## CONCLUSION

A key unresolved question for future work is the basis on which to distinguish new potential subtypes of autism. Since early cognitive impairments are linked to very early emergence of autism, *even if only for some of the early‐diagnosed cases*, then clarification of these relationships within a developmental *as well as* neurobiological perspective would be highly informative for future theoretical, research, and clinical work. The concept of (very) early low IQ is distinguished from higher‐IQ in later childhood, adolescence, and adulthood, as well as from delay in language or speech onset, on the basis of very early age and impairments in both non‐verbal as well as verbal abilities. Below 2 years of age, early low IQ is a unique subtype of children who will be diagnosed with autism in early childhood. Children with ASD within this subgroup (early low IQ or very early low IQ), are characterized by substantial motor delays, familial likelihood, increased chance of DN mutations in neurodevelopment‐associated genes, and increased brain size, in addition to impaired cognitive abilities. The state of the art in the field indicates that the etiology of these early‐diagnosed, very early low IQ ASD cases is different from later‐diagnosed, average or higher‐IQ cases, and from cases of school‐aged children with speech delay onset, some of whom may have intact fluid intelligence. Research studies are needed to answer new questions arising in order to understand more comprehensively how these different groupings differ in neurobiology. As much work remains to be done, in particular with regard to advancing health equity for all children, these distinctions can serve as guidance for the complex and growing investigation of cognitive abilities and ASD.


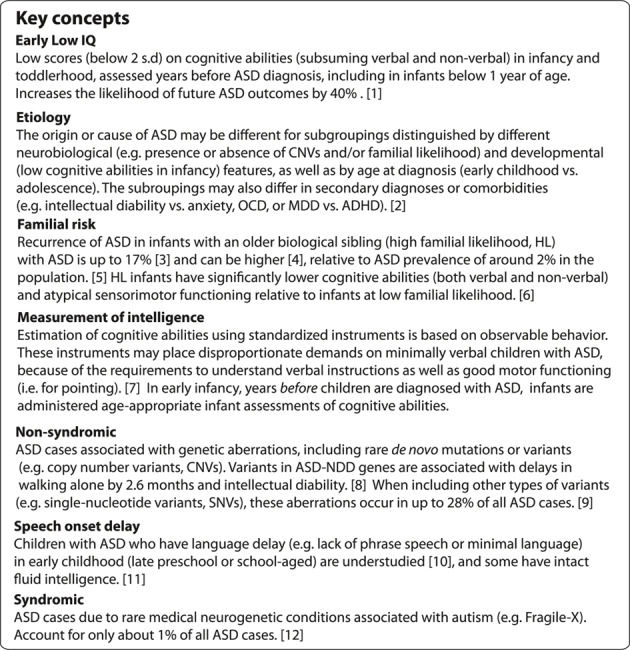


Note: References: [1] Denisova & Lin 2022; [2] Gandal 2016; [3] Palmer et al.,^2017^; [4] Ozonoff et al.,^2011^; [5] Maenner 2023; [6] Denisova & Zhao.,^2017^; [7] Dawson 2007; [8] Satterstrom et al.,^2020^; [9] Iossifov et al.,^2014^; [10] Tager‐Flusberg 2013; [11] Courchesne et al., 2015; [12] Geschwind 2011.

## AUTHOR CONTRIBUTIONS

**Kristina Denisova**: Conceptualization; funding acquisition; visualization; writing—original draft; writing—review & editing.

## CONFLICT OF INTEREST STATEMENT

The author declares no conflicts of interest with regard to the funding sources for this study. The Denisova lab gratefully acknowledges generous support from the SFARI under Award Number 62646 (PI: Kristina Denisova) and National Institute of Mental Health of the National Institutes of Health under Award Number R01MH121605 (PI: Kristina Denisova). The content is solely the responsibility of the author and does not necessarily represent the official views of the National Institutes of Health. The sponsors had no role in study design, in the collection, analysis, and interpretation of data, in the writing of this report, and in the decision to submit the manuscript for publication. The author is on the DEIA Committee at the Department of Psychology, City University of New York, Queens College. The author has declared that they have no competing or potential conflicts of interest.

## ETHICAL CONSIDERATIONS

No ethical approval was required for this review.

## Data Availability

Data sharing is not applicable to this article as no datasets were generated or analyzed during the current study. Figure 2 was generated based on Table S4 in Satterstrom et al., 2020.
